# The Science and Practice of Medicine

**Published:** 1867-01

**Authors:** 


THE SCIENCE AND PRACTICE OF MEDICINE. By WILLIAM AITKEN, M.D., Edin., Prof. of Pathology in the Army Medical School; Corresponding Member of the Royal Imperial Society of Physicians of Vienna, etc., etc., etc. In two volumes. From the fourth London edition, with additions by MEREDITH CLYMER, M.D., late Professor of the Institutes and Practice of Medicine in the University of New York, etc., etc. Philadelphia: LINDSAY & BLAKISTON. 1866.

We have received from the publishers, the first volume of this work, with a notice that the second volume will be through the press in a few days. We have only time and space now to say that the American publishers have issued the work in good style; and that it constitutes a very full and valuable treatise on practical medicine. All our readers who desire a very full summary of the present condition of practical medicine in Great Britain, will certainly find it in this work. When we have received the second volume, we will notice it more in detail. Price of complete work $12.00

